# Assessing the Categories and Factors Influencing Treatment Decision Conflict in Patients With Lumbar Disc Herniation: A Latent Profile Analysis

**DOI:** 10.1111/hex.70924

**Published:** 2026-10-17

**Authors:** Yuan Tian, Bing‐Lin Li, Ming‐Kui Shen, Shi‐Na Cheng, Xiao‐Wei Jia, Shao‐Hua Chen

**Affiliations:** ^1^ Spine and Joint Surgery Ward The Third People's Hospital of Henan Province Zhengzhou Henan China; ^2^ Henan Engineering Research Center of Precision Diagnosis and Treatment of Intervertebral Disc Disease Zhengzhou Henan China; ^3^ Nursing Department The Third People's Hospital of Henan Province Zhengzhou Henan China; ^4^ Department of Mini‐invasive Spinal Surgery The Third People's Hospital of Henan Province Zhengzhou Henan China; ^5^ School of Nursing Fujian Medical University Fuzhou Fujian China

**Keywords:** influencing factors, latent profile analysis, lumbar disc herniation, treatment decision

## Abstract

**Background:**

Lumbar disc herniation (LDH) represents a prevalent degenerative spinal disorder, characterized by severe pain and functional limitations that significantly diminish patients' quality of life. Despite the availability of conservative and surgical treatment options, patients frequently encounter considerable decisional conflict (DC). Current research often neglects the inherent heterogeneity within this patient population. To ensure that the research questions and outcome measures reflect patients' real‐world priorities, we involved patients with LDH in the study design phase. This study employs latent profile analysis (LPA) to identify distinct patterns in treatment decision‐making and examine their underlying factors, thereby providing a scientific basis for personalized clinical decision support.

**Methods:**

This cross‐sectional study involved 743 patients diagnosed with LDH. Patients and public were involved in refining the questionnaire content and validating the relevance of outcome measures. The data collection tools included a General Information Questionnaire, the Decisional Conflict Scale (DCS), the Health Belief Scale, and the Treatment Expectancy Questionnaire. Influential factors were identified through univariate analyses and multinomial logistic regression. LPA was used to identify latent profiles and delineate their unique characteristics.

**Results:**

Three distinct latent profiles emerged: the low‐DC group (23.55%), the moderate DC group (44.55%), and the high‐DC group (31.90%). Multinomial logistic regression analysis indicated that factors such as gender, marital status, educational attainment, type of medical insurance, employment status, annual household income, place of residence, and clinical history (including duration since onset and prior consultations) significantly influenced membership in these profiles. Additionally, treatment expectations and health beliefs were identified as critical psychological predictors of DC.

**Conclusion:**

Patients with LDH exhibit substantial heterogeneity in treatment DC, classified into low, moderate, and high‐conflict groups via latent profile analysis, with over 70% experiencing moderate to high DC. DC in patients with LDH is influenced by multidimensional factors, including sociodemographic characteristics, clinical features, health beliefs, and treatment expectations, with health beliefs and treatment expectations acting as core protective factors. These findings highlight the need to replace the one‐size‐fits‐all model with personalized decision‐support strategies for patients with LDH based on conflict profiles and individual characteristics.

**Abbreviations**
aBIC: adjusted Bayesian Information Criterion
AIC: Akaike information criterion
BLRT: Bootstrap‐based Likelihood ratio test
CI: confidence interval
CT: computed tomography
DC: decisional conflict
DCS: Decisional Conflict Scale
HBS: Health Belief Scale
LDH: lumbar disc herniation
LMR: Lo‐Mendell‐Rubin likelihood ratio test
LPA: latent profile analysis
MRI: magnetic resonance imaging
OR: odds ratio
TEX‐Q: Treatment Expectation Questionnaire

## 1 Background

Lumbar disc herniation (LDH) is defined as a limited displacement of disc material beyond the edge of the disc space, resulting in the compression or irritation of lumbar nerve roots and causing inflammatory reactions manifesting as pain and neurological dysfunction [1]. Low back pain, often attributed to LDH, constitutes the leading cause of disability worldwide [2], with a prevalence of LDH of about 7.62% [3]. In China, the prevalence of lumbar spine diseases varies between 4% and 11%, influenced by age and occupation [4]. The prevalence of LDH in the Chinese population increases with age [5], significantly impairing patient quality of life due to pain and dysfunction [6, 7].

Conservative and surgical treatments represent the main therapeutic options for LDH [8, 9]. Most patients experience improvement with conservative treatment over a duration of 6–12 weeks [10, 11]. Common conservative treatments include bed rest, medication, exercise therapy, epidural injections, and lumbar traction [12, 13]. Surgical treatment is recommended as the preferred option for severe pain unresponsive to conservative measures, signs of cauda equina syndrome, or progressive motor weakness in patients with a British Medical Research Council muscle strength rating of ≤ 3/5 [14, 15]. However, treatment choices are rarely absolute and necessitate comprehensive assessments of various influencing factors. Although conservative treatment is generally safer and better suited for patients with mild or acute conditions, its prolonged duration often results in delayed efficacy [16]. Surgical treatment may be more effective for patients with severe pain or functional limitations, but it also raises concerns about surgical risks, including intraoperative anaesthesia, potential nerve injuries, and postoperative recurrence [17]. This uncertainty may stem from diverse factors, including fear of surgery, efforts to understand the medical situation, costs associated with surgery and hospitalization, concerns about postsurgical dependency, and awareness of pain severity.

Decision‐making ambivalence frequently results in diagnostic delays and subsequent decisional regret, adversely affecting clinical outcomes and patient satisfaction. In this context, accurate treatment decisions are crucial for patient prognosis, yet contradictions within the decision‐making process, such as trade‐offs between efficacy and risk and between short‐term and long‐term benefits, may hinder patients' rational choices [18, 19]. Treatment decisions are not solely determined by disease severity but are influenced by multifaceted factors, resulting in decision conflict.

Decision conflict (DC) refers to the uncertainty or difficulty experienced by patients when choosing a risky or outcome‐based option [20]. Higher levels of DC can cause emotional distress, including anxiety and depression [21], while also contributing to delays in medical decision‐making, increasing regret, and heightening the risk of medical adverse events [22].

Previous studies have explored several factors contributing to DC, including demographic variables (age, gender, socioeconomic status), clinical characteristics (symptom duration, complications), and psychosocial factors [23, 24, 25]. Our previous study identified associations between demographic factors such as gender, marital status, educational level, insurance type, employment status, annual income, place of residence, the duration since onset, and first‐hospital level with treatment DC in patients with LDH [26]. However, existing research mostly focuses on the independent role of a single or a limited number of factors in DC, with insufficient in‐depth exploration of the internal dimensional characteristics of DC. This exploration is crucial for medical professionals to attain a comprehensive understanding of patients' treatment decision‐making dilemmas, guiding them toward formulating effective interventions. The Decision Conflict Scale (DCS) developed by O'Connor et al. [27] in 1995 contains five core dimensions, confirming that DC in patients with LDH is often not uniformly distributed but may be concentrated in a single or several dimensions, complicating the implementation of precise interventions in clinical practice. Thus, it is imperative to delineate the characteristics and influencing factors of different subtypes of decisional conflict through typing studies to provide a basis for targeted interventions. This study used a cross‐sectional survey to investigate the treatment decision‐making status of patients with LDH.

Latent profile analysis (LPA) is a robust model‐based probabilistic approach that relies on latent category theory to identify homogeneous subgroups from latent characteristics of research subjects, thereby capturing population heterogeneity [28]. LPA possesses unique methodological strengths for exploring DC subtypes in patients with LDH. It adopts multidimensional DCS scores as continuous indicator variables to objectively categorize heterogeneous patient populations into mutually exclusive latent subgroups based solely on empirical data fitting, rather than on subjective empirical judgement. Furthermore, LPA captures subtle internal structural differences and latent multidimensional heterogeneity of DC across patient subgroups, enabling precise classification of clinical DC subtypes and providing refined, stable, and reliable evidence for identifying subgroup‐specific determinants and developing targeted and individualized clinical intervention strategies. To ensure that the research questions and outcome measures reflect the real‐world priorities and decision‐making experiences of patients with LDH, we involved patients in the study design phase. Their input helped refine the content and relevance of the data collection instruments. Therefore, this study aims to investigate the factors influencing DC in patients with LDH, and use LPA to identify the latent category types of patient DC. The findings would provide a scientific basis for developing individualized treatment plans that enhance the quality of treatment decisions.

## 2 Methods

This cross‐sectional study used a convenience sampling method to recruit participants from January to July 2025 in a tertiary hospital located in Zhengzhou City, Henan Province, China. We report patient and public involvement in this study according to the GRIPP2 Short Form reporting checklist (Supporting Material 1).

### 2.1 Study Participants

Participants included patients who attended rehabilitation, minimally invasive spine surgery, and traditional Chinese medicine outpatient and inpatient clinics during the study period. Inclusion criteria encompassed the following: LDH confirmed by lumbar spine magnetic resonance imaging (MRI) and computed tomography (CT) examinations; lumbar MRI and CT plain scans showing LDH, prolapse, or release; age of 18 years; and voluntary participation in the study. Exclusion criteria included patients with communication disorders. Written informed consent was obtained from all participants.

### 2.2 Data Collection

Before the survey, three data collection team members underwent standardized training. A unified guidance language was used when administering the questionnaires, wherein investigators explained the purpose and content of the study to eligible patients, obtained their cooperation, and secured signed informed consent. Data were collected using self‐administered questionnaires. For participants facing literacy or visual barriers, neutral and non‐suggestive assistance was provided by investigators. The data questionnaire was primarily administered in electronic format, supplemented by paper versions. To ensure completeness and accuracy of the electronic version of the questionnaire, each IP address was restricted to one submission, with all entries needing completion before submission. After filling out the paper version of the questionnaire, it was collected on‐site, where investigators checked for omissions or errors, prompting participants to verify and correct the responses as needed.

A total of 749 questionnaires were collected, yielding 743 valid responses, resulting in an effective response rate of 99.20%. For individual missing scale items (missing rate < 1%), the mean‐filling method was used to ensure data integrity.

#### 2.2.1 Demographics

Structured questionnaires were used to collect demographic information, including gender, marital status, age, education level, insurance type, employment status, per capita annual household income, and place of residence.

#### 2.2.2 Measurement of DC

Treatment DC was assessed using the Chinese version of the DCS, originally compiled by O'Connor et al. [28] and adapted cross‐culturally by Li [29]. The scale comprises 16 items across five subscales: information adequacy (3 items), value clarity (3 items), support (3 items), uncertainty (3 items), and effective decision‐making (4 items). Responses are scored on a 5‐point Likert scale (0 = *strongly agree* to 4 = *strongly disagree*), with a total score calculated by multiplying the mean item score by 25. The score range spans 0–100 points, with 0–25 indicating low conflict, 26–50 moderate conflict, and 51–100 high conflict. Higher scores indicate more severe DCs. The internal consistency reliability of the Chinese version of the scale is good (Cronbach's *α* = 0.897).

#### 2.2.3 Measurement of Health Beliefs

Health beliefs were measured using the revised Chinese version of the Health Belief Scale (HBS). The original scale was translated by Yuan [30] and serves as a common tool for assessing health beliefs. Ji et al. [31] verified its cross‐cultural adaptation and validity, establishing good test‐retest reliability (test‐retest reliability = 0.889), semi‐reliability (half‐reliability = 0.936), and internal consistency reliability (Cronbach's *α* = 0.967). The scale comprises 48 items and includes five subscales: personal health beliefs, perceived implementation ability, perceived sense of control, perceived resource utilization, and perceived threat. Scoring operates on a 5‐point Likert scale (1 = *very weak* to 5 = *very strong*), with higher total scores indicating stronger health beliefs.

#### 2.2.4 Measurement of Treatment Expectancy

The Chinese version of the Treatment Expectation Questionnaire (TEX‐Q) was developed by Shedden‐Mora et al. [32, 33] and performed by Yang [34] at the Hamburg‐Eppendorf University Medical Center in Germany. The questionnaire consists of 15 items covering six dimensions: treatment benefit, positive affect, adverse events, negative affect, treatment process, and behaviour control. Scoring was based on an 11‐point Likert scale (0–10 points). Following reverse scoring of the Injury Expectancy subscale (items 7–11), an overall mean score is calculated, with higher scores indicating more positive overall treatment expectations. The reliability of the Chinese version of the questionnaire is excellent reliability (overall Cronbach's *α* = 0.903; Cronbach's *α* = 0.842–0.924 for each subscale; semi‐reliability = 0.958).

### 2.3 Statistical Analysis

Rversion 4.4.2 and the mclust package were used for data processing and statistical analyses. Logical checks and verification of completeness were conducted on retrieved questionnaires, discarding invalid questionnaires with more than 10% missing key information. Scale items with a missing rate of < 1% were imputed using the mean‐filling method within the respective subscales. Categorical variables were described using frequency (*n*) and percentage (%), with differences in distribution between groups analysed using the *χ* ^2^ test. Continuous variables were described using mean ± standard deviation and median (interquartile range), with normality assessed using the Shapiro–Wilk test.

Variables with fulfilling normal distribution and uniform variance assumptions were compared between groups using one‐way analysis of variance, whereas non‐normally distributed variables were analysed using the Mann–Whitney *U* test or Kruskal–Wallis *H* test as appropriate.

Internal consistency reliability was evaluated using Cronbach's *α* coefficient and the standardized Cronbach's *α* coefficient. The strength and direction of the association between core variables were explored using Pearson's correlation analysis. The number of categories was gradually expanded from a one‐category model to five‐category model, selecting optimal models based on Akaike Information Criterion (AIC), Bayesian Information Criterion (BIC), sample‐corrected BIC (aBIC), entropy index, Mendel–Rubin likelihood ratio test (LMR), Bootstrap‐based likelihood ratio test (BLRT), and category representativeness (≥ 8%). Variable with *p* < 0.05 in univariate analysis were considered independent variables, whereas the DC category identified by LPA served as the dependent variable. A multivariate logistic regression model with forward selection was constructed to assess the independent risk factors that contributed to membership within the DC categories. Results were reported as odds ratios (OR) and corresponding 95% confidence intervals (CI).

### 2.4 Patient and Public Involvement

We recruited eight patients with LDH representing diverse demographic backgrounds, insurance types, and disease durations as research partners. Through offline meetings—supported by plain‐language materials and one‐on‐one discussions—they participated in scale revision, questionnaire pilot testing, and interpretation of subgroup findings. Patient feedback clarified ambiguous scale items and optimized survey guidance, increasing the valid response rate to 99.20%. Their clinical insights also enriched the interpretation of key influencing factors such as marital status and disease duration, thereby enhancing data authenticity and clinical applicability. However, attendance was inconsistent, with two of the eight members attending only one of the two sessions.

## 3 Results

### 3.1 Basic Characteristics of the Study Participants

A total of 743 patients with LDH were included in this study. In total, 469 (63.1%) participants were males and 274 females (36.9%), and most of the participants (*n* = 504, 67.8%) were married. Majority of the participants were aged > 60 years (40.9%), followed by those aged 45–59 years (26.2%) and those aged < 44 years (32.8%). Educational attainment was predominantly high school (36.3%), followed by undergraduate degree holders (22.3%). Regarding the types of medical insurance, basic medical insurance for urban and rural residents constituted the largest category (51.5%). Employment status showed that 59.8% were working, while the annual income groups were relatively similar for < 30,000 and 30,000–80,000 yuan, comprising 28.5% and 29.7%, respectively. Most participants lived in urban settings (59.4%) (Table 1).

**Table 1:** Univariate analysis of potential categories of DCS.

Variables	Attributes	Total (*n* = 743)	Low (*n* = 175)	Middle (*n* = 237)	High (*n* = 331)	*χ* ^2^/*F*	*p*
Gender	Male	469 (63.12%)	90 (51.43%)	151(63.71%)	228 (68.88%)	15.033	< 0.001
	Female	274 (36.88%)	85 (48.57%)	86 (36.29%)	103 (31.12%)		
Marital	No	239 (32.17%)	25 (14.29%)	83 (35.02%)	131 (39.58%)	34.858	< 0.001
	Yes	504 (67.83%)	150 (85.71%)	154 (64.98%)	200 (60.42%)		
Age, years	< 44	244 (32.84%)	64 (36.57%)	88 (37.13%)	92 (27.79%)	9.662	0.047
	45–59	195 (26.24%)	51 (29.14%)	56 (23.63%)	88 (26.59%)		
	> 60	304 (40.92%)	60 (34.29%)	93 (39.24%)	151 (45.62%)		
Education	Primary	81 (10.9%)	38 (21.71%)	18 (7.59%)	25 (7.55%)	63.946	< 0.001
	Junior	143 (19.25%)	40 (22.86%)	43 (18.14%)	60 (18.13%)		
	High	270 (36.34%)	32 (18.29%)	82 (34.6%)	156 (47.13%)		
	School	83 (11.17%)	28 (16%)	29 (12.24%)	26 (7.85%)		
	Bachelor	166(22.34%)	37 (21.14%)	65 (27.43%)	64 (19.34%)		
Insurance	Urban Employee Basic Medical Insurance	383 (51.55%)	61 (34.86%)	132 (55.7%)	190 (57.4%)	31.989	< 0.001
	Urban Resident Basic Medical Insurance	327 (44.01%)	109 (62.29%)	91 (38.4%)	127 (38.37%)		
	Self‐funded care	33 (4.44%)	5 (2.86%)	14 (5.91%)	14 (4.23%)		
Employment	Unemployed	122 (16.42%)	69 (39.43%)	26 (10.97%)	27 (8.16%)	93.808	< 0.001
	Working	444 (59.76%)	70 (40%)	162 (68.35%)	212 (64.05%)		
	Retired	177 (23.82%)	36 (20.57%)	49 (20.68%)	92 (27.79%)		
Annual Income	Low‐income	212 (28.53%)	83 (47.43%)	63 (26.58%)	66 (19.94%)	69.552	< 0.001
	Lower‐middle‐class	221 (29.74%)	61 (34.86%)	69 (29.11%)	91 (27.49%)		
	Solidly middle‐class	149 (20.05%)	21 (12%)	49 (20.68%)	79 (23.87%)		
	Upper‐class	161 (21.67%)	10 (5.71%)	56 (23.63%)	95 (28.7%)		
Residence	non‐City	302 (40.65%)	89 (50.86%)	84 (35.44%)	129 (38.97%)	10.607	0.005
	City	441 (59.35%)	86 (49.14%)	153 (64.56%)	202 (61.03%)		
Duration of disease	< 3 months	215 (28.94%)	48 (27.43%)	69 (29.11%)	98 (29.61%)	103.983	< 0.001
	3–6 months	160 (21.53%)	34 (19.43%)	54 (22.78%)	72 (21.75%)		
	6–12 months	213 (28.67%)	14 (8%)	74 (31.22%)	125 (37.76%)		
	> 1 year	155 (20.86%)	79 (45.14%)	40 (16.88%)	36 (10.88%)		
Having seen a doctor	No	121 (16.29%)	19 (10.86%)	48 (20.25%)	54 (16.31%)	6.519	0.038
	Yes	622 (83.71%)	156 (89.14%)	189 (79.75%)	277 (83.69%)		
First Visit	3 Outp	281 (37.82%)	79 (45.14%)	93 (39.24%)	109 (32.93%)	20.341	0.002
	3 Hosp	97 (13.06%)	25 (14.29%)	34 (14.35%)	38 (11.48%)		
	2 Outp	259 (34.86%)	61 (34.86%)	76 (32.07%)	122 (36.86%)		
	2 Hosp	106 (14.27%)	10 (5.71%)	34 (14.35%)	62 (18.73%)		
The form of the first visit and the level of hospital	Community	67 (9.02%)	4 (2.29%)	18 (7.59%)	45 (13.6%)	232.707	< 0.001
	Physio	98 (13.19%)	3 (1.71%)	33 (13.92%)	62 (18.73%)		
	2Outp+	38 (5.11%)	5 (2.86%)	19 (8.02%)	14 (4.23%)		
	2Hosp+	74 (9.96%)	10 (5.71%)	30 (12.66%)	34 (10.27%)		
	3Outp+	225 (30.28%)	17 (9.71%)	83 (35.02%)	125 (37.76%)		
	3Hosp+	241 (32.44%)	136 (77.71%)	54 (22.78%)	51 (15.41%)		

*Note:* Annual income: Per capita annual household income; Upper‐class: > ¥150,000 (≈$20,985); Solidly middle‐class: ¥80,000 ~ 150,000 (≈$11,216 ~ $20,985); Lower‐middle‐class: ¥30,000–¥80,000 (≈$4,19–11,216); Low‐income: < ¥30,000 (≈$4,197).

Abbreviations: 3Hosp, hospitalization in tertiary hospital; 3 Outp, outpatient at tertiary hospital; 2 Hosp, hospitalization in secondary hospital; 2 Outp, outpatient at secondary hospital.

### 3.2 Latent Profile Analysis and Classification of Characteristics of Treatment DC

The five dimensions of the DCS total score (information adequacy, value clarity, decision support, decision certainty, and decision validity) served as explicit indicators for latent profile analysis, fitting 1–5 latent category models (Table 2). The AIC, BIC, aBIC values decreased gradually with increasing number of categories, whereas the entropy values peaked within the four‐category model (0.926). However, some categories had less than 5% representation (37 participants), which failed to meet the minimum sample size requirements. The AIC, BIC, and aBIC values for the three‐category model remained reasonable, with an acceptable entropy value of 0.793. Both LMR and BLRT tests showed *p* < 0.001, indicating that the three‐category models were significantly better than the two category model. Additionally, proportions across the three‐category model exceeded 8% (23.5% for low group, 31.9% for medium group, and 44.5% for high group), and the average probability of category attribution exceeding 0.84 (Table 3), indicating good classification stability. Therefore, the three‐category model was determined as the optimal classification scheme: 23.6% (175 cases) in the low‐DC group, 31.9% (237 cases) in the medium DC group, and 44.5% (331 cases) in the high‐DC group. Distinction between categories was good (self‐attribution probabilities ranging from 0.841 to 0.978) (Table 3). Figure 1 shows the scores of the three categories across each dimension of psychological capital.

**Table 2:** Fitting indicators for each potential category of DCS.

C	AIC	BIC	aBIC	Entropy	LMR (*p*)	BLRT (*p*)	Class probability
1	8898.463	−8990.677	8927.169	—	—	—	—
2	8670.774	−8790.652	8708.093	0.885	< 0.001	< 0.001	0.717, 0.283
3	8588.291	−8735.834	8634.222	0.793	< 0.001	< 0.001	0.319, 0.445, 0.236
4	8523.674	−8652.773	8563.863	0.926	< 0.001	< 0.001	0.168, 0.038, 0.624, 0.17
5	8385.55	−8588.421	8448.705	0.844	< 0.001	< 0.001	0.168, 0.312, 0.316, 0.171, 0.032

Abbreviations: AIC, Akaike Information Criterion; aBIC, adjusted Bayesian Information Criterion; BIC, Bayesian Information Criterion; BLRT, bootstrap likelihood ratio test; LMR, aBIo‐Mendell‐Rubinadiusted likelihood Ratio test.

**Table 3:** Mean probability for each potential category.

	*C* = 1	*C* = 2	*C* = 3
*C* = 1	0.841	0.099	0.060
*C* = 2	0.093	0.907	0.000
*C* = 3	0.022	0.000	0.978

Abbreviations: *C* = 1, low‐decisional conflict group; *C* = 2, middle‐decisional conflict group; *C* = 3, high‐decisional conflict group.

**Figure 1:**
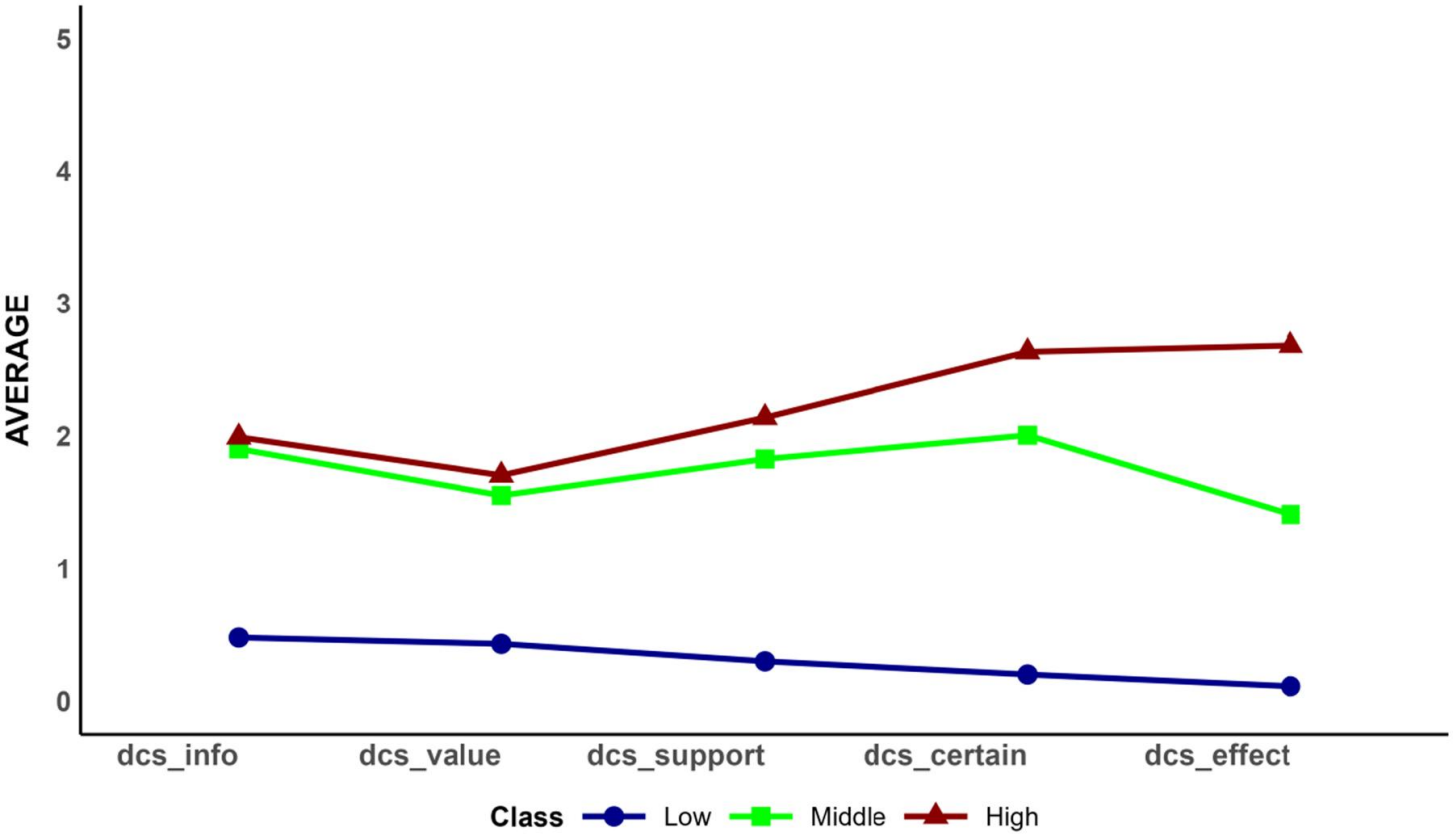
Potential characteristics of Treatment Decision Conflict. Figure 1 Latent profiles of treatment decision conflict among patients with lumbar disc herniation. The three identified patterns were low (*n* = 175, 23.55%), moderate (*n* = 332, 44.55%), and high (*n* = 237, 31.90%) decisional conflict groups.

### 3.3 Univariate Analysis of Treatment DC

The results showed that gender, marital status, education level, medical insurance type, employment status, annual income, area of residence, symptom onset, whether patients had sought medical treatment, first‐visit institution type, access to medical services, health trust scores, and treatment expectancy scores were significantly correlated with the total score of decisional conflict (*p* < 0.05). All variables that showed significant between‐group differences were included in subsequent analyses as covariates (Table 2).

### 3.4 Multivariate Analysis Affecting the Classification and Attribution of Treatment DC

Subgroups based on total DCS scores (low, medium, and high groups) were used as dependent variables, whereas variables with *p* < 0.05 in univariate analyses were treated as independent variables. Multivariate logistic regression analysis using the forward selection method was used to identify the significant independent factors influencing differences between groups. Analysis revealed that being married, obtaining a secondary school education, possessing resident medical insurance, demonstrating a willingness to visit a hospital above level 3, exhibiting high treatment expectations, and holding high health beliefs were more likely to be classified into the high‐DC group (all *p* < 0.05).

Notably, willingness to seek medical treatment and health beliefs displayed significant independent effects on the differences between the high and middle DC groups (all *p* < 0.05). The above factors were all protective factors, suggesting that an increase of these factors may reduce the probability of patients being classified into the high total DCS score, suggesting that higher levels of such factors led to reduced DC and facilitated classification into the middle group. Taking the low group (C1) as the reference category, multivariate logistic regression analysis showed that outpatient visits, treatment expectations, health beliefs, and medical institutions visits in level 2 hospitals had significant independent effects on the difference between the middle and low groups (all *p* < 0.05, Table 4).

**Table 4:** Multivariate logistic regression analysis of DCS.

Predictors		Middle & High			Middle & Low			High & Low		
		*OR*	*CI*	*p*	*OR*	*CI*	*p*	*OR*	*CI*	*p*
(Intercept)		7.88	0.50–133.88	0.146	0.00	0.00–0.00	< 0.001	0.00	0.00–0.00	< 0.001
Gender	Female	1.04	0.70–1.54	0.860	0.75	0.35–1.58	0.449	1.41	0.55–3.60	0.473
Marital	Yes	0.90	0.62–1.31	0.588	1.15	0.46–2.93	0.766	3.85	1.41–11.30	0.011
Education	Junior	0.87	0.40–1.89	0.731	0.42	0.11–1.53	0.189	1.87	0.38–9.64	0.443
	High	1.14	0.55–2.35	0.715	0.73	0.19–2.86	0.649	1.04	0.23–4.91	0.959
	School	0.63	0.26–1.52	0.309	1.03	0.25–4.24	0.965	10.01	1.74–65.81	0.012
	Bachelor	0.78	0.36–1.70	0.536	0.87	0.22–3.50	0.845	2.12	0.45–10.56	0.348
Insurance	BMI/NCMS	0.88	0.60–1.30	0.532	1.08	0.48–2.39	0.849	2.93	1.13–8.03	0.031
	ADE/CMI	0.55	0.24–1.26	0.156	0.83	0.09–6.18	0.868	1.03	0.06–13.41	0.986
Employment	Working	1.11	0.57–2.13	0.766	0.57	0.21–1.55	0.266	0.32	0.09–1.14	0.079
	Retired	1.43	0.70–2.94	0.324	0.75	0.24–2.34	0.623	0.33	0.07–1.38	0.131
Annual Income	Lower‐middle‐class	1.34	0.80–2.25	0.267	1.07	0.45–2.54	0.880	1.16	0.39–3.52	0.787
	Solidly middle‐class	1.22	0.70–2.12	0.481	0.51	0.17–1.49	0.217	1.31	0.34–5.15	0.692
	Upper‐class	0.98	0.56–1.71	0.951	0.76	0.19–2.81	0.685	1.12	0.21–5.54	0.888
Residence	City	0.90	0.61–1.31	0.579	0.84	0.38–1.84	0.654	0.94	0.37–2.47	0.904
Duration of disease	3–6 months	0.82	0.49–1.35	0.428	1.56	0.59–4.23	0.370	1.42	0.46–4.42	0.541
	6–12 months	1.01	0.65–1.59	0.950	0.39	0.11–1.28	0.129	0.16	0.04–0.60	0.009
	> 1 year	0.88	0.47–1.65	0.692	1.19	0.48–2.95	0.699	0.54	0.15–1.83	0.333
Having seen a doctor	Yes	1.05	0.65–1.69	0.834	1.62	0.64–4.18	0.309	3.21	0.96–11.69	0.066
First visit	3Hosp	0.85	0.47–1.56	0.602	1.12	0.42–3.03	0.829	2.68	0.68–11.03	0.164
	2Outp	0.99	0.63–1.56	0.972	4.63	1.79–12.82	0.002	3.81	1.22–13.06	0.026
	2Hosp	1.27	0.74–2.21	0.390	0.99	0.25–3.71	0.989	1.18	0.28–4.84	0.816
Form of the first visit and the level of hospital	Physio	0.64	0.30–1.34	0.244	0.65	0.07–5.66	0.698	2.57	0.17–34.47	0.479
	2Outp+	0.32	0.12–0.82	0.019	1.28	0.18–9.21	0.804	3.77	0.26–58.08	0.333
	2Hosp+	0.37	0.17–0.80	0.013	3.99	0.72–24.82	0.121	15.87	1.98–147.77	0.012
	3Outp+	0.55	0.28–1.04	0.071	3.14	0.64–17.89	0.173	3.14	0.48–23.26	0.246
	3Hosp+	0.48	0.23–0.97	0.045	7.86	1.67–44.84	0.013	38.67	5.58–340.71	< 0.001
TEX		1.03	0.79–1.34	0.828	1.61	1.11–2.39	0.014	2.17	1.30–3.84	0.005
HBS		0.99	0.98–1.00	0.013	1.06	1.04–1.07	< 0.001	1.06	1.04–1.08	< 0.001
Current symptoms		0.81	0.61–1.07	0.144	1.44	0.81–2.61	0.214	1.43	0.78–2.66	0.254
Medical institutions previously visited		1.25	1.05–1.49	0.011	0.50	0.30–0.80	0.005	0.42	0.26–0.67	< 0.001
Previous treatments received		1.21	0.89–1.67	0.230	1.03	0.54–1.95	0.933	0.34	0.15–0.72	0.007
Observations		568			412			506		
*R* ^2^ Tjur		0.097			0.001			0.792		

Abbreviations: HB, health belief; *R* ^2^ Tjur, Tjur's coefficient of discrimination (*D*)/Tjur's Pseudo *R* ^2^; TEX, treatment expectations.

## 4 Discussion

### 4.1 Classification Characteristics and Intervention Strategies for LDH Patient Groups

This cross‐sectional study included 743 patients with LDH. The findings indicated that 76.4% of patients experienced moderate to high DC regarding treatment, whereas the remaining 23.5% reported low DC, suggesting that difficult treatment decision‐making is prevalent among patients with LDH and represents a common clinical issue that cannot be ignored. Treatment DC is a major clinical problem encountered by patients with LDH during diagnosis, treatment, and option selection. It is associated with the complexity of treatment regimens, individual cognitive differences, and prognostic uncertainty, and may directly affect treatment adherence, rehabilitation outcomes, and quality of life. The findings are consistent with previous studies [35] and further confirm the high prevalence and complexity of treatment DC among these patients.

Further LPA revealed that treatment DC in patients with LDH reflects significant heterogeneity, categorized into three profiles: low conflict (23.5%), moderate conflict (31.9%), and high conflict (44.5%). Significant differences were observed among all three groups across all five dimensions of the DCS. The high‐conflict group accounted for the largest proportion, displaying the highest scores on information acquisition, values clarity, decision support, decision certainty, and decision effectiveness, particularly in decision certainty and effectiveness. Patients in this group showed substantial barriers in understanding and evaluating treatment information, difficulty weighing benefits and risks, insufficient perceived social support, high decision uncertainty, and low self‐efficacy, representing the highest priority population for clinical intervention. For these patients, targeted one‐on‐one information support, structured decision aids, and multidimensional emotional and social support should be provided to reduce uncertainties, enhance confidence, and improve decision certainty and effectiveness [35].

Patients in the moderate conflict group recorded moderate scores across most dimensions yet showed a marked deficit in decision effectiveness. This indicates that although their information acquisition was relatively adequate, they lacked confidence in implementing their decisions. This group represents a transitional population at risk of progressing to high conflict without timely intervention. Interventions for this group should focus on consolidating knowledge and improving decision‐making self‐efficacy through group health education, peer modelling, and decision‐making skills training to enhance their sense of control.

Conversely, the low‐conflict group had the lowest scores across all dimensions, characterized by sufficient information, clear values, adequate social support, and strong confidence in their decision‐making abilities. Maintaining favourable decision‐making status for these patients necessitates regular follow‐up to monitor post‐decision adaptations and encouraging their roles as peer supporters.

The three‐profile classifications identified in this study further validate the heterogeneity of treatment DC in patients with LDH. These results advocate for a stratified, personalized decision‐support approach as opposed to a universal strategy in clinical practice. For patients with moderate and high DC, targeted interventions can address cognitive biases, alleviate decision‐making challenges, and improve decision‐making quality, ultimately providing a theoretical basis and practical direction for optimizing clinical outcomes and rehabilitation in patients with LDH.

### 4.2 Factors Influencing Latent Profiles in Treatment Decision‐Making

This study found that patients who were not married were 3.85 times more likely to belong to the high‐conflict group compared with married patients (OR = 3.85, 95% CI: 1.41–11.30, *p* = 0.011), suggesting an association between marital status and a significantly higher level of treatment decision‐making conflict. Consistent with the findings by Hobbs [36], stable romantic relationships promote critical emotional and informational support. Such support may be correlated with helping patients clarify value preferences and reduce uncertainty in medical decisions, thus alleviating decision‐making conflict. Family members of married patients play an important role in decision‐making, especially in Asian populations with high family participation. In contrast, patients who are not married are more susceptible to decision dilemmas due to a lack of such support, prompting clinicians to encourage family participation in diagnosis and treatment decisions to mitigate negative emotional impacts through enhanced social support [37].

Given the cross‐sectional nature of this investigation, the observed relationships should be interpreted as associations rather than definitive causal effects. The impact of educational attainment on decision‐making conflict is not a simple linear relationship; rather, it exhibits unique gradient characteristics. Patients with a senior high school education demonstrated a higher likelihood of belonging to the high‐conflict group with a total DCS score of 45.97 ± 17.92. It is speculated that their intermediate comprehension of health information, combined with insufficient medical knowledge, may be associated with a tendency toward an ‘information anxiety zone’ when confronted with extensive medical information, which correlates with elevated DC. Conversely, individuals with a primary school education or less were more likely to belong to the low‐conflict group (DCS = 29.57 ± 23.60), and this pattern may be linked to their reliance on authoritative medical advice, which minimizes decision autonomy and hastens decisional processes. The bachelor's degree and above group, with higher health literacy, may be associated with achieving ‘sufficient informed consent’ through professional literature retrieval and in‐depth communication with medical staff, improving decision certainty and favouring classification within the low‐conflict group [38]. This contrasts with the hypothesis that ‘individuals with higher education have stronger decision‐making ability and lower conflict’ [39].

As a key economic factor, medical insurance type was found to be associated with significant differences in decision‐making conflict levels. Patients under the urban and rural resident basic medical insurance (URBMI) and those relying on self‐paid or commercial medical insurance (ADE/CMI) were more likely to be in the high‐conflict group, whereas patients covered by the basic medical insurance or the new rural cooperative medical system (BMI/NCMS) were more likely to be in the low‐conflict group. The total DCS scores reflected this, with scores among URBMI patients (44.50 ± 20.29) significantly higher than those among patients in the BMI/NCMS group (35.36 ± 23.38) (*p* < 0.001), consistent with the findings by Pandrangi [40]. Variations in insurance coverage and reimbursement ratios may correlate with treatment choices, which could contribute to higher uncertainty among URBMI patients. Notably, patients with self‐paid or commercial insurance had the highest DCS scores (46.45 ± 22.20), and this observation may be associated with the substantial economic burden of treatment costs that correlates with decision‐making anxiety [41].

Employment status and annual household income also exhibited associations with decision‐making conflict through potential links to economic security and perceived time costs. Employed patients (DCS = 44.13 ± 19.73) and those with annual household income over 150,000 yuan (DCS = 49.87 ± 15.38) were more likely to be in the high‐conflict group, which may be related to pressures regarding work‐treatment balance and higher opportunity costs. Conversely, unemployed, retired, and low‐income patients (annual income < 30,000 yuan) tended to be in the low‐conflict group, possibly because of their singular decision‐making priorities or lack of work‐related concerns, which may reduce the need to make trade‐offs [42, 43].

Urban patients (DCS = 42.66 ± 21.80) were more likely to fall within the high‐conflict group compared with their non‐urban counterparts (37.50 ± 22.57, *p* = 0.002). The abundance of medical resources coupled with fragmented, low‐quality health information in urban areas cause cognitive confusion, whereas non‐urban patients have limited choices and rely on local medical advice, reducing decision hesitancy [44].

### 4.3 Impact of Onset Duration and Type of Institution on Patients' Decision‐Making Conflicts

The time from first onset to medical consultation exhibited a distinct non‐linear correlation with patients with LDH' treatment DC profiles. Patients presenting with a 6–12 month onset were most likely to fall into the high‐DC group, whereas those with over 1 year of illness tended to be in the low‐DC group. The former group had the highest DCS score (47.16 ± 14.39), whereas lower scores were recorded by the latter group (27.60 ± 27.83), with an extremely significant intergroup difference (*p* < 0.001). The 6–12 month interval since symptom onset was observed to be associated with higher odds for the high‐DC group compared with the low‐DC group (OR = 0.16, 95% CI: 0.04–0.60, *p* = 0.009). This pattern aligns with clinical guidelines for LDH [15, 45]: within 3 months of symptom onset, most patients receive conservative treatment with a clear decision direction, which correlates with low DC [41]. Between three to 6 months, poor conservative treatment outcomes prompt surgical consideration yet with doubts about timing, leading to gradually rising DC and moderate DC classification. At 6–12 months, anxiety from ineffective conservative treatment and concerns over surgical risks create dual pressure, plunging patients into a dilemma between delayed recovery and surgical complications and pushing DC to a peak (high‐DC group); for patients with over 1 year of onset, chronic pain fosters rational treatment expectations or a fixed conservative treatment model, which may be associated with marked reductions in decision doubts and lead to low DC [37, 41].

The type of first‐visit medical institution directly correlates with DC profiles via diagnosis and treatment resource allocation as well as the quality of doctor‐patient communication. Patients first consulting at secondary hospital inpatients were most likely to be high‐DC, whereas those first visiting tertiary hospital outpatients tended to be low‐DC, with respective DCS scores of 46.21 ± 17.13 and 38.21 ± 23.92 (*p* = 0.0254). First consulting secondary hospital outpatients was identified as a factor associated with elevated risk for the high‐DC group (OR = 3.81, 95% CI: 1.22–13.06, *p* = 0.026). Secondary hospitals have orthopaedic resources between tertiary and community hospitals: they can recommend surgery but lack tertiary‐level multidisciplinary collaboration, failing to provide comprehensive evaluations and plan interpretations. This leaves patients underinformed on the core surgical decisions, which may correlate with exacerbated DC. Despite high patient volume, tertiary hospital outpatients benefit from experienced specialists who clearly explain surgical indications and contraindications, helping patients narrow treatment options and reduce hesitation (low‐DC status). Community hospitals predominantly focus on conservative treatment with limited options, yet their patients had a relatively high DCS score (50.51 ± 15.31) in this study, confirming that non‐specialized resources fail to meet diverse patient decision needs and are easily associated with high DC levels.

### 4.4 Influence of Personal Health Beliefs and Treatment Expectations on the Classification of Treatment Decision‐Making Conflict in Patients With LDH

Patients with higher levels of personal health beliefs and treatment expectations were more likely to belong to the low treatment decision‐making conflict group, and both were observed to be protective factors associated with reduced DC and correlated with patient classification within the low‐conflict group. High health beliefs and high treatment expectations may help patients understand their own conditions more clearly, clarify their value orientation for treatment, and reduce biases in the process of receiving and understanding diagnosis and treatment‐related information [46, 47, 48]. At the same time, they may be associated with improved patient self‐efficacy in treatment decision‐making, strengthening their judgements and reducing hesitation and anxiety when facing treatment options, thereby correlating with a lower level of decision‐making conflict. In contrast, patients with weak health beliefs or low treatment expectations have a vague understanding of their conditions, lacking confidence in predicting treatment effects. They are easily disturbed by external information in diagnosis and treatment decision‐making and struggle to develop clear preferences for treatment options, rendering them more susceptible to moderate or high‐conflict categorization [49]. This result is supported by correlation analyses in this study, indicating that the total score of personal health beliefs and the average score of treatment expectations were significantly negatively correlated with the total score of decision‐making conflict. This clearly indicates that personal health beliefs and treatment expectations act as not merely influencing factors but also as core internal factors that may contribute to the classification of patients' decision‐making conflict. In clinical practice, improving patients' health beliefs and guiding them to establish reasonable and positive treatment expectations may facilitate the transformation of patients into the low decision‐making conflict group while simultaneously improving the quality of their treatment decision‐making.

### 4.5 Research Limitations

Adopting a cross‐sectional design limits the understanding of characteristics related to patients' decision‐making conflict and the correlations with relevant influencing factors at a single time point, thereby failing to elucidate the dynamic progression and key turning points of decision‐making conflict during the course of disease diagnosis and treatment. In addition, the study samples were only collected from a single province in China, and consisted of patients with LDH who were actively involved in treatment and recruited via convenience sampling from hospitals, leading to a relatively homogeneous sample, which consequently limits the generalizability of the research findings. The patient research partners were also recruited from the same single‐centre setting, and the small group size further limited the diversity of perspectives obtained. Since the data were based on patients' self‐reports, sample bias may be introduced. Notably, all primary variables in the current study were collected via self‐administered questionnaires, which may introduce substantial response bias and measurement errors. Self‐reported data are susceptible to recall bias, social desirability effects, and differences in individual subjective perception, which may distort the true status of patients' treatment DC and related psychological characteristics. Such inherent limitations of questionnaire‐based self‐report measures may confound the observed associations and affect the reliability of the study findings. Despite these limitations, the involvement of patients enriched the patient‐centredness of the questionnaires and added real‐world relevance to the interpretation of our findings.

Future research should leverage longitudinal follow‐up designs to monitor long‐term patterns among patients, aiming to reveal the dynamic evolution law and core turning points of treatment decision‐making conflict in patients with LDH. Meanwhile, the geographical coverage of samples and the scope of population inclusion should be expanded to further enhance the generalizability and clinical applicability of the findings. Moreover, future studies could adopt multisource data collection methods, combining objective clinical assessment indicators with subjective questionnaire reports, to reduce the potential impact of response bias and improve the robustness of the study findings. Future PPI efforts should also consider recruiting a larger and more diverse group of patient partners and establishing regular communication mechanisms to ensure sustained and representative engagement throughout the research process.

## 5 Conclusions

Health beliefs and treatment expectations were key psychological protective factors, with stronger beliefs and more positive expectations associated with lower DC. The three DC subtypes established in this study yield important clinical implications and can be applied as a practical grading tool to assist clinicians in rapidly assessing patients' decision‐making status in routine clinical practice. Patients in the low‐conflict subgroup, who maintain stable health beliefs and positive treatment expectations, merely require conventional standardized health guidance. In contrast, patients in the moderate‐ and high‐conflict subgroups, which accounted for the majority of the sample, are more likely to develop negative disease perceptions, treatment hesitation, and pronounced decisional uncertainty. This classification can support clinicians in implementing tiered shared decision‐making strategies tailored to different patient groups. Specifically, high‐risk patients with severe DC can receive individualized cognitive intervention to clarify personalized treatment regimens and prognostic information and to correct disease‐related misconceptions. For patients with moderate DC, regular standardized health education combined with dynamic follow‐up is conducive to preventing the deterioration of decision‐making hesitation. This subtype‐based assessment and intervention framework enables precise identification of high‐risk populations, standardizes clinical decision‐support processes, and optimizes patient–provider shared decision‐making, thereby reducing DC in LDH treatment. These findings not only highlight the heterogeneity of treatment‐related decision‐making in patients with LDH but also underscore the core value of targeted patient health education in reducing DC. Implementing stratified health education for different DC subgroups can help patients establish sound health beliefs and treatment expectations by correcting cognitive biases and delivering evidence‐based disease and treatment information, thereby reducing information asymmetry and decisional uncertainty. Such interventions can enhance patients' decision‐making participation and quality, providing an important practical basis for subsequent personalized interventions and optimized clinical outcomes.

## Author Contributions

**Yuan Tian:** conceptualization, methodology, data curation, formal analysis, validation, investigation, visualization, project administration, resources, writing – review and editing, writing – original draft. **Bing‐Lin Li:** data curation, validation, investigation, project administration. **Ming‐Kui Shen:** methodology, software, data curation, validation, funding acquisition, resources. **Shi‐Na Cheng:** conceptualization, investigation, data curation. **Xiao‐Wei Jia:** investigation, data curation. **Shao‐Hua Chen:** writing – original draft, writing – review and editing, project administration, formal analysis, conceptualization.

## Ethics Statement

This study was conducted in accordance with the Declaration of Helsinki and approved by the Ethics Committee of The Third People's Hospital of Henan Province (approval No. 2025SZSYLCYJ0404).

## Consent

Written informed consent was obtained from all individual participants included in the study.

## Conflicts of Interest

The authors declare no conflicts of interest.

## Patient or Public Contribution (PPIE)

We recruited eight patients with LDH representing diverse demographic backgrounds, insurance types, and disease durations as research partners.

## Supporting information


Supporting File

## Data Availability

The data that support the findings of this study are available from the corresponding author upon reasonable request.

## References

[1] C. A. Hincapié , D. Kroismayr , L. Hofstetter , et al., “Incidence of and Risk Factors for Lumbar Disc Herniation With Radiculopathy in Adults: A Systematic Review,” European Spine Journal 34 (2025): 263–294, 10.1007/s00586-024-08528-8. PMC11754359 39453541

[2] M. L. Ferreira , K. De Luca , L. M. Haile , et al., “Global, Regional, and National Burden of Low Back Pain, 1990–2020, Its Attributable Risk Factors, and Projections to 2050: A Systematic Analysis of the Global Burden of Disease Study 2021,” Lancet Rheumatology 5 (2023): e316–e329, 10.1016/S2665-9913(23)00098-X. PMC10234592 37273833

[3] N. N. Knezevic , K. D. Candido , J. W. S. Vlaeyen , J. Van Zundert , and S. P. Cohen , “Low Back Pain,” Lancet 398 (2021): 78–92, 10.1016/S0140-6736(21)00733-9. 34115979

[4] S. Ji , Q. Shao , Y. Wang , and J. Liu , “Correction: Efficacy Comparison Between Minimally Invasive and Conventional Surgery for Lumbar Disc Herniation in Chinese Han Population: A Meta‐Analysis,” Spinal Cord 55 (2017): 626, 10.1038/sc.2015.240. 26679374

[5] P. Yin , J. L. Qi , Y. N. Liu , et al., “Report on Disease Burden in China From 2005 to 2017. Chinese,” Circulation Journal 34, no. 12 (2019): 1145–1154.

[6] L. R. Vialle , E. N. Vialle , J. E. Suárez Henao , and G. Giraldo , “Lumbar Disc Herniation,” Revista Brasileira de Ortopedia 45 (2015): 17–22, 10.1016/S2255-4971(15)30211-1. PMC4799068 27019834

[7] Chinese Orthopaedic Association of Orthopaedic Rehabilitation Group ., “Clinical Practice Guideline for Diagnosis and Treatment of Lumbar Disc Herniation,” Chinese Journal of Orthopaedics 40 (2020): 477–487, 10.3760/cma.j.cn121113-20200402-00213.

[8] U. M. Ahn , N. U. Ahn , J. M. Buchowski , E. S. Garrett , A. N. Sieber , and J. P. Kostuik , “Cauda Equina Syndrome Secondary to Lumbar Disc Herniation: A Meta‐Analysis of Surgical Outcomes,” Spine 25 (2000): 1515–1522, 10.1097/00007632-200006150-00010. 10851100

[9] C. Liu , G. E. Ferreira , C. Abdel Shaheed , et al., “Surgical Versus Non‐Surgical Treatment for Sciatica: Systematic Review and Meta‐Analysis of Randomised Controlled Trials,” BMJ 381 (2023): e070730, 10.1136/bmj-2022-070730. PMC10498296 37076169

[10] N. Kögl , O. Petr , W. Löscher , U. Liljenqvist , and C. Thomé , “Lumbar Disc Herniation,” Deutsches Ärzteblatt international 121 (2024): 440–448, 10.3238/arztebl.m2024.0074. PMC11465477 38835174

[11] K. W. Rickers , P. H. Pedersen , T. Tvedebrink , and S. P. Eiskjær , “Comparison of Interventions for Lumbar Disc Herniation: A Systematic Review With Network Meta‐Analysis,” The Spine Journal 21 (2021): 1750–1762, 10.1016/j.spinee.2021.02.022. 33667683

[12] C. S. Bailey , P. Rasoulinejad , D. Taylor , et al., “Surgery Versus Conservative Care for Persistent Sciatica Lasting 4 to 12 Months,” New England Journal of Medicine 382 (2020): 1093–1102, 10.1056/NEJMoa1912658. 32187469

[13] F. Dai , Y. X. Dai , H. Jiang , P. F. Yu , and J. T. Liu , “Non‐Surgical Treatment With XSHHD for Ruptured Lumbar Disc Herniation: A 3‐Year Prospective Observational Study,” BMC Musculoskeletal Disorders 21 (2020): 690, 10.1186/s12891-020-03723-2. PMC7574183 33076896

[14] H. Haro , S. Ebata , G. Inoue , et al., “Japanese Orthopaedic Association (JOA) clinical practice guidelines on the management of lumbar disc herniation, third edition—secondary publication,” Journal of Orthopaedic Science 27 (2021): 31–78, 10.1016/j.jos.2021.07.028. 34865915

[15] D. S. Kreiner , S. W. Hwang , J. E. Easa , et al., “An Evidence‐Based Clinical Guideline for the Diagnosis and Treatment of Lumbar Disc Herniation With Radiculopathy,” Spine Journal 14 (2014): 180–191, 10.1016/j.spinee.2013.08.003. 24239490

[16] Y. Wang , G. Dai , L. Jiang , and S. Liao , “The Incidence of Regression After the Non‐Surgical Treatment of Symptomatic Lumbar Disc Herniation: A Systematic Review and Meta‐Analysis,” BMC Musculoskeletal Disorders 21 (2020): 530, 10.1186/s12891-020-03548-z. PMC7419225 32778091

[17] R. Clark , R. P. Weber , and L. Kahwati , “Surgical Management of Lumbar Radiculopathy: A Systematic Review,” Journal of General Internal Medicine 35 (2020): 855–864, 10.1007/s11606-019-05476-8. PMC7080952 31713029

[18] T. Rätsep , A. Abel , and Ü. Linnamägi , “Patient Involvement in Surgical Treatment Decisions and Satisfaction With the Treatment Results After Lumbar Intervertebral Discectomy,” European Spine Journal 23 (2014): 873–881, 10.1007/s00586-013-3104-y. PMC3960447 24249555

[19] B. Bozyiğit , M. D. Yurtluk , H. Kına , P. Akbarov , and A. S. Apaydın , “Gender Disparities in Decision‐Making and Outcomes in Lumbar Spine Surgery: A Retrospective Analysis,” Medical Records 7, no. 2 (2025): 450–453, 10.37990/medr.1636552.

[20] A. N. Heirman , D. P. de Kort , J. F. Petersen , et al., “Decisional Conflict in Patients With Advanced Laryngeal Carcinoma: A Multicenter Study,” Laryngoscope 134 (2024): 3604–3610, 10.1002/lary.31336. 38366759

[21] J. Yuan , F. Xu , H. Ren , M. Chen , and S. Feng , “Distress and Its Influencing Factors Among Chinese Patients With Incidental Pulmonary Nodules: A Cross‐Sectional Study,” Scientific Reports 14 (2024): 1189, 10.1038/s41598-023-45708-w. PMC10786871 38216579

[22] J. Sweeney , C. Tichnell , S. Christian , et al., “Characterizing Decision‐Making Surrounding Exercise in ARVC: Analysis of Decisional Conflict, Decisional Regret, and Shared Decision‐Making,” Circulation: Genomic and Precision Medicine 16 (2023): e004133, 10.1161/CIRCGEN.123.004133. PMC10729899 38014565

[23] M. Wosny , S. Aeppli , S. Fischer , T. Peres , C. Rothermundt , and J. Hastings , “Factors Guiding Clinical Decision‐Making in Genitourinary Oncology,” Cancer Medicine 13 (2024): 70304, 10.1002/cam4.70304. PMC11494402 39435678

[24] M. Andkhoie , D. Meyer , and M. Szafron , “Factors Underlying Treatment Decision‐Making for Localized Prostate Cancer in the U.S. and Canada: A Scoping Review Using Principal Component Analysis,” Canadian Urological Association Journal 13 (2018): E220, 10.5489/cuaj.5538. PMC6667325 30472985

[25] A. S. Apaydın , M. Güneş , M. Yana , C. C. Aksoy , I. Saraçoğlu , and C. Akıncı , “The Turkish Validity and Reliability of Hospital for Special Surgery‐Lumbar Spine Surgery Expectations Survey,” Journal of Turkish Spinal Surgery 35, no. 2 (2024): 55–60, 10.4274/jtss.galenos.2024.95530.

[26] Y. Tian , S. H. Chen , R. P. Song , et al., “The Impact of Health Beliefs on Treatment Decision Conflict Through Treatment Expectations Mediation in Patients With Lumbar Disc Herniation,” Scientific Reports 15 (2025): 42802, 10.1038/s41598-025-27025-6. PMC12663123 41315529

[27] A. M. O'Connor , H. Llewellyn‐Thomas , and A. Flood , “Decision Conflict Scale: Development and Evaluation of a Tool to Measure Decisional Uncertainty,” Medical Decision Making 19 (1999): 25–30.

[28] Q. Guo , Y. Wang , R. Zheng , et al., “Death Competence Profiles and Influencing Factors Among Novice Oncology Nurses: A Latent Profile Analysis,” BMC Nursing 23 (2024): 939, 10.1186/s12912-024-02641-1. PMC11660589 39707293

[29] Y. Li , “Research on the Construction and Application of a Treatment Decision‐Making Aid Plan for Patients With Early Primary Liver Cancer.” Dissertation (Second Military Medical University, 2017), 10.1016/S0140-6736(25)01917-8.

[30] L. H. Wang , J. H. Peng , X. P. Zhang , et al., “Mediating Effect of HBs Between Health Knowledge and Health Behavior in Patients With Hypertension and Stroke,” Chinese Journal of Behavioral Medicine and Brain Sciences 26 (2017): 847–851, 10.3760/cma.j.issn.1674-6554.2017.09.016.

[31] S. Ji and Y. Hui , “Cross‐Cultural Debugging and Reliability and Validity Evaluation of Health Belief Scale,” Medical Innovation of China 9 (2013): 139–141.3, 10.3969/j.issn.1674-4985.2013.09.075.

[32] J. Alberts , B. Löwe , M. A. Glahn , et al., “Development of the Generic, Multidimensional Treatment Expectation Questionnaire (TEX‐Q) Through Systematic Literature Review, Expert Surveys and Qualitative Interviews,” BMJ Open 10 (2020): e036169, 10.1136/bmjopen-2019-036169. PMC7440833 32819942

[33] M. C. Shedden‐Mora , J. Alberts , K. J. Petrie , et al., “The Treatment Expectation Questionnaire (TEX‐Q): Validation of a Generic Multidimensional Scale Measuring Patients' Treatment Expectations,” PLoS One 18 (2023): e0280472, 10.1371/journal.pone.0280472. PMC9870103 36689398

[34] L. N. Yang , The Sinicization of the Treatment Expectation Questionnaire (tEX‐Q) and Its Application in Colorectal Cancer Patients. Dissertation. (Nanchang: Nanchang University, 2023).

[35] C. Ribeiro , P. Pamplona , and A. K. Simonds , “Advance Care Planning in Patients With Respiratory Failure,” European Respiratory Review 33 (2024): 240120, 10.1183/16000617.0120-2024. PMC11558536 39537243

[36] K. E. Pecanac , R. L. Brown , and H. B. Kremsreiter , “Decisional Conflict During Major Medical Treatment Decision‐Making: A Survey Study,” Journal of General Internal Medicine 36 (2021): 55–61, 10.1007/s11606-020-06125-1. PMC7431116 32808213

[37] P. Mitropoulou , N. Grüner‐Hegge , J. Reinhold , and C. Papadopoulou , “Shared Decision Making in Cardiology: A Systematic Review and Meta‐Analysis,” Heart 109 (2022): 34–39, 10.1136/heartjnl-2022-321050. 36007938

[38] R. Hwang , S. Lambrechts , H. Liu , et al., “Decisional Conflict Among Patients Considering Treatment Options for Lumbar Herniated Disc,” World Neurosurgery 116 (2018): e680–e690, 10.1016/j.wneu.2018.05.068. 29783012

[39] K. Laryionava , D. Hauke , P. Heußner , W. Hiddemann , and E. C. Winkler , “Often Relatives Are the Key […]”—Family Involvement in Treatment Decision Making in Patients With Advanced Cancer Near the End of Life,” Oncologist 26 (2021): e831–e837, 10.1002/onco.13557. PMC8100569 33037846

[40] V. C. Pandrangi , N. F. Farrell , J. C. Mace , K. Y. Detwiller , T. L. Smith , and M. Geltzeiler , “Perceived Financial Insecurity Impacts Healthcare Decision‐Making Among Patients With Sinusitis,” Laryngoscope 131 (2021): 2403–2412, 10.1002/lary.29561. 33851727

[41] M. J. Metz , M. A. Veerbeek , C. M. van der Feltz‐Cornelis , E. de Beurs , and A. T. F. Beekman , “Decisional Conflict in Mental Health Care: A Cross‐Sectional Study,” Social Psychiatry and Psychiatric Epidemiology 53 (2018): 161–169, 10.1007/s00127-017-1467-9. 29209746

[42] R. Burch , P. Rizzoli , and E. Loder , “The Prevalence and Impact of Migraine and Severe,” Headache: The Journal of Head and Face Pain 61 (2021): 60–68, 10.1111/head.14024. 33349955

[43] G. S. Hobbs , M. B. Landrum , N. K. Arora , et al., “The Role of Families in Decisions Regarding Cancer Treatments,” Cancer 121 (2015): 1079–1087, 10.1002/cncr.29064. PMC4368490 25708952

[44] W. B. Brinkman , “Physicians' Shared Decision‐Making Behaviors in Attention‐Deficit/Hyperactivity Disorder Care,” Archives of Pediatrics & Adolescent Medicine 165 (2011): 1013–1019, 10.1001/archpediatrics.2011.154. PMC3759988 22065181

[45] M. Haiqing , Z. Feifei , and C. Siyi , “Expert Consensus on the Implementation Process of Percutaneous Lumbar Endoscopic Surgery for Accelerated Rehabilitation Surgery,” Chinese Journal of Bone and Joint Surgery 12 (2019): 641–651.

[46] T. Rakhshani , E. Razeghi , S. M. Kashfi , A. Kamyab , and A. Khani Jeihooni , “The Effect of Educational Intervention Based on Health Belief Model on Colorectal Cancer Screening Behaviors,” BMC Public Health 24 (2024): 1640, 10.1186/s12889-024-19180-8. PMC11188256 38898503

[47] X. Li , Q. Lu , J. Li , and M. Zhang , “Correlation Between Exercise Compliance After Discharge and Health Belief in Patients Underwent Hip Arthroplasty: A Longitudinal Study,” Chinese Journal of Nursing 54 (2019): 1180–1184, 10.3761/j.issn.0254-1769.2019.08.012.

[48] L. Xiao , M. Peng , Y. Liu , and L. Zhang , “Information, Deliberation, and Decisional Control Preferences for Participation in Medical Decision‐Making and Its Influencing Factors Among Chinese Cancer Patients,” Health Expectations 24 (2021): 1725–1736, 10.1111/hex.13312. PMC8483183 34309132

[49] L. B. Oswald , R. Kasimer , K. Rappazzo , A. J. Fought , D. F. Penson , and A. K. Morgans , “Patient Expectations of Benefit From Systemic Treatments for Metastatic Prostate Cancer,” Cancer Medicine 9 (2020): 980–987, 10.1002/cam4.2783. PMC6997053 31840434

