# Supplementary material for: Assessing the Categories and Factors Influencing Treatment Decision Conflict in Patients With Lumbar Disc Herniation: A Latent Profile Analysis
Source: Health Expect. 2026 Oct 17;29(5):e70924. doi: 10.1111/hex.70924 (PMC13676030; doi:10.1111/hex.70924)
Supplement: Supplementary file 1 [file HEX-29-e70924-s001.pdf]

Supplemental File: GRIPP2 Form Short Checklist

Adapted from: Staniszewska S, Brett J, Simera I, Seers K, Mockford C, Goodlad S, et al. GRIPP2 reporting checklists: tools to improve reporting of patient and public involvement in research. *BMJ*. 2017;358:j3453

**Section and topic**	**Item**	**Reported section**
Aim	Report the aim of PPI (patient and public involvement) in the study.	Methods
Methods	Provide a clear description of the methods used for PPI in the study.	Methods
Study results	Outcomes—Report the results of PPI in the study, including both positive and negative outcomes.	Interpretation
Discussion and conclusion	Outcomes—Comment on the extent to which PPI influenced the study overall. Describe positive and negative effects.	Methods, Interpretation
Reflections/critical perspective	Comment critically on the study, reflecting on the things that went well and those that did not, so others can learn from this experience.	Interpretation
